# Supplementary material for: Fungal Biomarkers in Traditional Starter Determine the Chemical Characteristics of Turbid Rice Wine from the Rim of the Sichuan Basin, China
Source: Foods. 2023 Jan 30;12(3):585. doi: 10.3390/foods12030585 (PMC9914865; doi:10.3390/foods12030585)
Supplement: Supplementary file 1 [file foods-12-00585-s001.zip › foods-2183536-supplementary.pdf]

Supplementary Materials

Fungal Biomarkers in Traditional Starter Determine the Chemical Characteristics of Turbid Rice Wine from the Rim of the Sichuan Basin, China

Lanchai Chen [1,2,3], Wenliang Xiang [1,2,3,*], Xuemei Liang [1,4], Junyu Liu [1], Haoyu Zhu [1], Ting Cai [1,2,3], Qing Zhang [1,2,3] and Jie Tang [1,2,3]

[1] School of Food and Bioengineering, Xihua University, Chengdu 610039, China

[2] Key Laboratory of Food Microbiology of Sichuan, Xihua University, Chengdu 610039, China

[3] Chongqing Key Laboratory of Speciality Food Co-Built by Sichuan and Chongqing, Xihua University, Chengdu 610039, China

[4] Sichuan Vocational School of Commerce, Chengdu 611731, China

* Correspondence: biounicom@mail.xhu.edu.cn; Tel.: +86-28-8772-0552

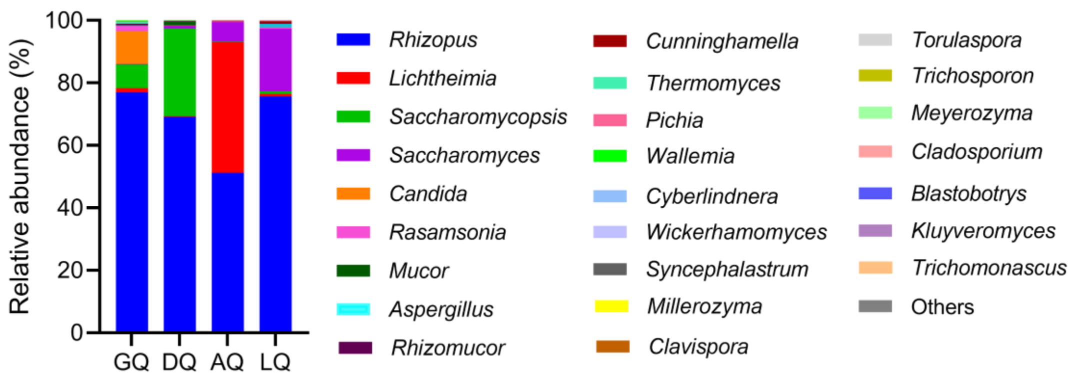

Figure S1. Fungal communities in 4 regional Qu varieties at genus level. Results are shown as the mean from three biological replicates.

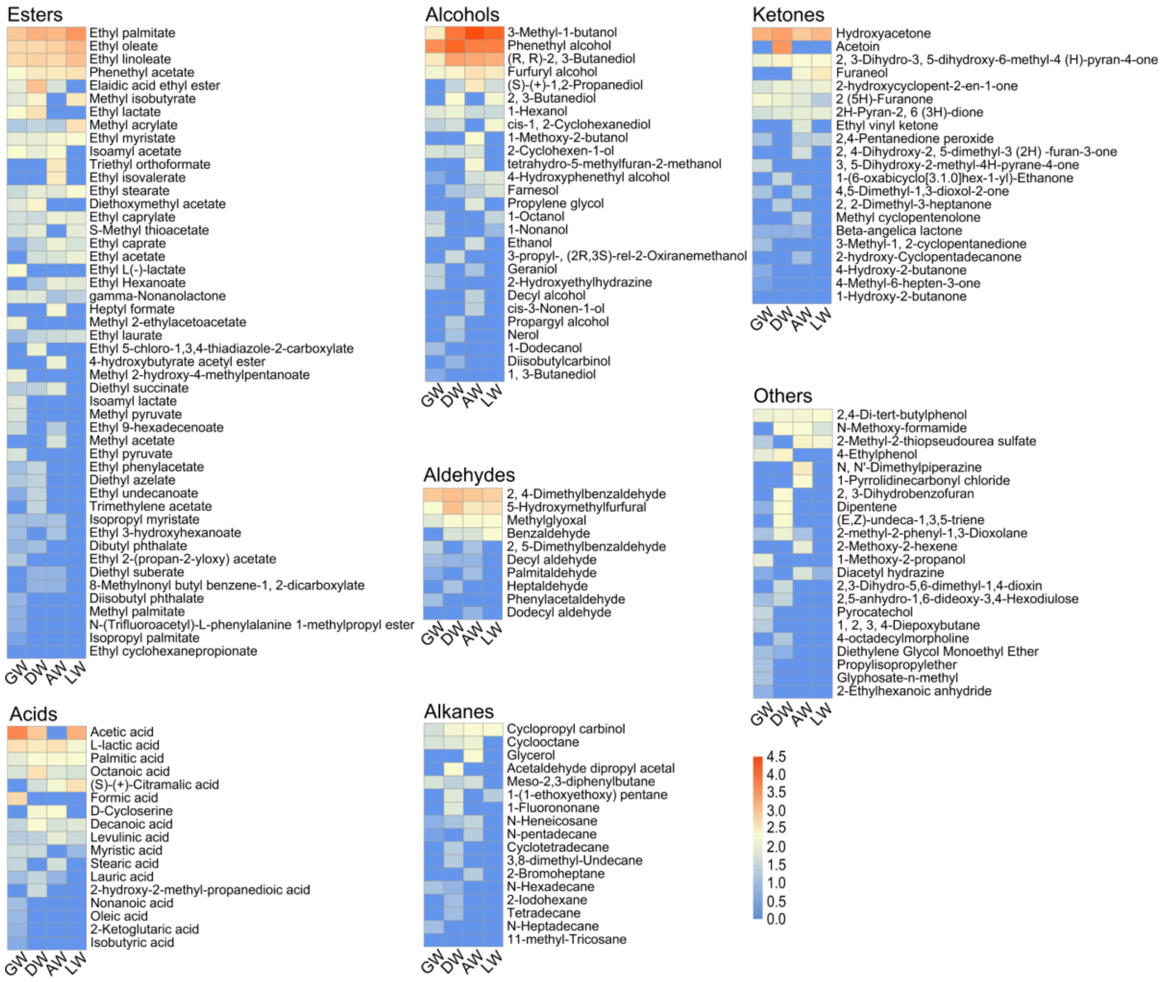

Figure S2. Heatmap of volatile compound contents in rice wine fermented using different Qu. The contents of volatile compound were processed logarithmically.

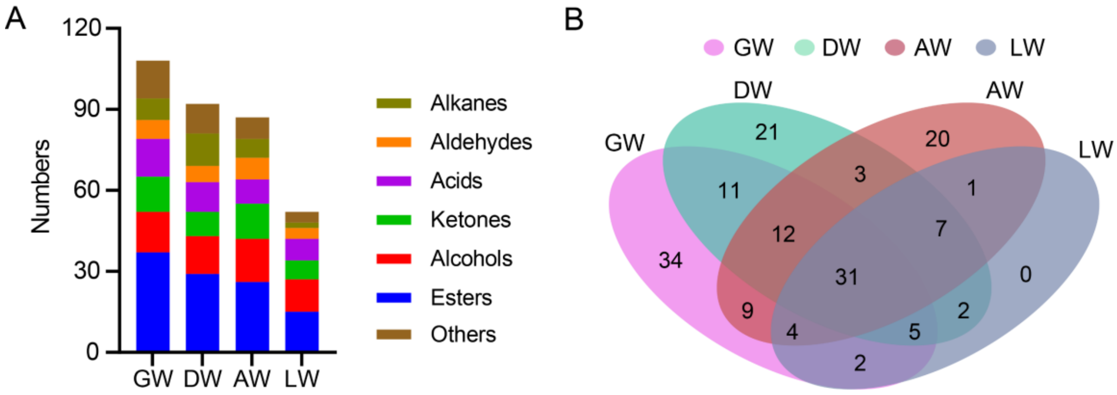

Figure S3. Types of volatile compound in rice wine fermented using different Qu. A. Stack graph of types of volatile compound classified into esters, alcohols, ketones, acids, aldehydes, alkanes, and other compounds; B. Venn diagram of types of volatile compound in different rice wine samples.

Table S1 The correlation coefficient (|r| ≥ 0.7, $p < 0.05$) between fungi and important volatile compounds with ≥ 1 mg/L of total volatile compounds through all TRWs.

Fungal species	Acetic acid	L-lactic acid	3-Methyl-1-butanol	Phenethyl alcohol	(R, R)-2, 3-Butanediol	2, 4-Dimethylbenzaldehyde	5-Hydroxymethylfurfural	Ethyl palmitate	Ethyl oleate	Ethyl linoleate	Acetoin
**GQ biomarkers**											
Rhizopus arrhizus	0.75	–	−0.87	–	–	–	–	–	−0.78	–	–
Candida glabrata	–	–	–	–	–	–	–	−0.74	–	–	–
Rhizomucor pusillus	0.72	–	–	−0.87	−0.95	–	−0.88	–	–	–	−0.75
Thermomyces lanuginosus	0.77	–	–	−0.90	−0.92	–	−0.80	–	–	–	−0.76
Wallemia sebi	–	–	−0.84	–	–	–	–	–	−0.88	–	–
Lichtheimia corymbifera	–	–	–	–	–	0.78	–	–	−0.83	–	–
Pichia kudriavzevii	0.84	–	–	−0.71	–	–	–	–	–	–	–
**DQ biomarkers**											
Saccharomycopsis fibuligera	–	–	−0.75	–	–	–	–	–	–	–	0.73
Mucor indicus	–	–	–	–	–	–	–	–	–	–	0.76
**AQ biomarkers**		–									
Lichtheimia ramosa	–	–	–	–	–	–	–	–	–	–	−0.75
**LQ biomarkers**											
Rhizopus microspores	–	−0.78	–	–	–	–	–	0.87	–	0.91	–
Saccharomyces cerevisiae	–	–	0.76	–	–	–	–	0.77	0.93	0.78	–
**Non−biomarkers**											
Aspergillus niger	–	−0.91	–	–	–	–	–	0.86	–	0.89	–
Aspergillus penicillioides	–	–	−0.80	–	–	–	–	−0.74	−0.83	–	–
Rhizomucor miehei	–	–	−0.86	–	–	0.75	–	–	−0.73	–	–
Cyberlindnera fabianii	−0.72	–	–	–	0.73	–	–	–	–	–	–
Diutina rugosa	–	–	–	–	–	0.75	–	–	–	–	–
Saccharomycopsis malanga	–	–	–	–	0.74	–	0.82	–	–	–	0.90
Syncephalastrum racemosum	–	–	–	–	−0.75	–	–	–	–	–	–
Clavispora lusitaniae	−0.78	–	–	0.76	0.72	–	–	–	–	–	–
Millerozyma farinosa	–	–	–	−0.81	−0.70	–	–	–	–	–	–
Syncephalastrum monosporum	–	–	–	–	–	–	–	–	–	–	–

Note: –, |r| < 0.7 or $p \geq 0.5$ or no correlation.

Table S2 The correlation coefficient (|r| ≥ 0.7, $p < 0.05$) between fungi and important volatile compounds with < 1 mg/L of total volatile compounds through all TRWs.

Fungal species	< 1 mg/L and ≥ 0.1 mg/L																						< 0.1 mg/L and ≥ 0.01 mg/L						
	Octanoic acid	(S)-(+)-Citramalic acid	D-Cycloserine	(S)-(+)-1,2-Propanediol	cis-1, 2-Cyclohexanediol	2-Cyclohexen-1-ol	4-Hydroxyphenethyl alcohol	Methylglyoxal	Benzaldehyde	Cyclooctane	Methyl isobutyrate	Methyl acrylate	Ethyl myristate	Isoamyl acetate	Ethyl stearate	S-Methyl thioacetate	Ethyl acetate	Diethyl succinate	Furaneol	2 (5H)-Furanone	N-Methoxy-formamide	2-Methyl-2-phenyl-1,3-dioxolane	Stearic acid	Lauric acid	Farnesol	Propylene glycol	1-Octanol	2, 5-Dimethylbenzaldehyde	N-Heneicosane
**GQ biomarkers**																													
Rhizopus arrhizus	–	−0.79	–	−0.92	–	–	–	–	–	–	–	–	–	–	–	–	–	–	−0.71	0.78	–	–	–	–	−0.73	–	–	–	–
Candida glabrata	–	–	–	–	–	–	–	–	−0.70	–	–	–	–	–	−0.76	–	–	–	–	–	–	–	–	–	–	–	–	0.76	–
Rhizomucor pusillus	–	–	–	–	–	–	–	−0.90	–	–	–	–	–	–	–	–	–	–	–	–	−0.87	–	–	–	–	–	0.72	–	–
Thermomyces lanuginosus	−0.73	–	−0.71	–	–	–	–	−0.94	–	–	–	–	–	–	–	–	–	–	–	–	−0.92	–	–	–	–	–	0.82	–	–
Wallemia sebi	–	−0.86	–	−0.87	–	–	–	–	−0.85	–	–	–	−0.83	–	–	–	−0.80	–	−0.80	0.87	–	–	–	–	−0.85	–	–	–	–
Lichtheimia corymbifera	–	−0.79	–	–	–	–	−0.89	–	−0.73	–	–	–	−0.89	–	–	–	−0.71	–	−0.90	0.83	–	0.75	–	0.77	−0.79	–	–	–	–
Pichia kudriavzevii	−0.71	–	−0.83	–	–	–	–	–	–	−0.70	–	–	–	–	–	–	–	–	–	–	−0.80	–	–	–	–	–	–	–	−0.80
**DQ biomarkers**																													
Saccharomycopsis fibuligera	–	–	–	−0.77	–	–	−0.73	–	–	–	–	–	–	–	–	–	–	–	−0.71	–	–	–	–	–	–	–	–	–	–
Mucor indicus	–	–	–	–	–	–	–	–	–	–	–	–	–	–	–	–	–	–	–	–	–	–	–	–	–	−0.71	–	–	–

**AQ biomarkers**																													
Lichtheimia ramosa	–	–	–	–	–	–	–	–	–	–	–	–	–	–	–	–	–	–	–	–	–	–	0.85	–	–	0.82	–	–	–
**LQ biomarkers**																													
Rhizopus microspores	–	–	–	–	0.83	−0.81	–	–	0.82	−0.81	0.82	0.91	–	−0.86	0.95	0.81	–	–	–	–	–	–	−0.71	–	–	−0.82	–	−0.86	–
Saccharomyces cerevisiae	–	0.97	–	0.76	–	–	0.75	–	0.86	–	–	–	0.87	−0.75	0.73	–	0.91	–	0.91	−0.95	–	–	–	–	0.89	–	–	–	–
**Non−biomarkers**																													
Aspergillus niger	–	–	–	–	0.88	−0.75	–	–	0.70	–	0.88	0.91	–	−0.92	0.91	0.85	–	–	–	–	–	–	−0.86	–	–	−0.86	–	−0.86	–
Aspergillus penicillioides	–	−0.84	–	−0.84	–	–	–	–	−0.71	–	–	–	–	–	–	–	−0.71	–	−0.75	0.80	–	–	–	–	−0.88	–	–	–	–
Rhizomucor miehei	–	−0.74	–	−0.78	–	–	−0.81	–	–	–	–	–	−0.82	–	–	–	–	–	−0.83	0.78	–	–	–	–	–	–	–	–	–
Cyberlindnera fabianii	–	–	–	–	–	–	–	–	–	–	–	–	–	–	–	–	–	–	–	–	0.75	–	–	–	–	–	–	–	–
Diutina rugosa	–	–	–	–	–	–	−0.83	–	–	–	–	–	−0.73	–	–	–	–	–	−0.75	–	–	–	–	–	–	–	–	–	–
Saccharomycopsis malanga	–	–	–	–	–	–	–	–	–	–	–	–	–	–	–	–	–	–	–	–	–	–	–	–	–	–	–	–	–
Syncephalastrum racemosum	–	–	–	–	–	–	–	–	–	–	–	–	–	–	–	–	–	–	–	–	–	–	–	–	–	–	–	–	–
Clavispora lusitaniae	–	–	0.85	–	–	–	–	–	–	0.71	–	–	–	–	–	–	–	–	–	–	0.79	–	–	–	–	–	–	–	–
Millerozyma farinosa	–	–	–	–	–	–	–	−0.83	–	–	–	–	–	–	–	–	–	–	–	–	−0.72	–	–	–	–	–	–	0.72	–
Syncephalastrum monosporum	–	–	–	–	–	–	–	–	–	–	–	–	–	–	–	–	–	–	–	–	–	–	–	–	–	–	–	0.71	–

Note: −, |r| < 0.7 or $p \geq 0.5$ or no correlation.

Table S3 The correlation coefficient (|r| ≥ 0.7, $p < 0.05$) between fungi and the unique important volatile compounds in respective TRW.

Fungal species	GW				DW		AW												
	Formic acid	Methyl 2-ethylacetoacetate	Methyl pyruvate	1-Methoxy-2-propanol	Ethyl 5-chloro-1,3,4-thiadiazole-2-carboxylate	Acetoin	1-Methoxy-2-butanol	Tetrahydro-5-methylfuran-2-methanol	Glycerol	Triethyl orthoformate	Ethyl isovalerate	Heptyl formate	4-hydroxybutyrate acetyl ester	Methyl acetate	Ethyl vinyl ketone	2, 4-Dihydroxy-2, 5-dimethyl-3 (2H) -furan-3-	N, N'-Dimethylpiperazine	1-Pyrrolidinecarbonyl chloride	2-Methoxy-2-hexene
**GQ biomarkers**																			
Rhizopus arrhizus	0.75	0.73	0.73	0.75	–	–	–	–	−0.71	–	−0.71	–	–	–	–	–	–	–	−0.71
Candida glabrata	0.76	0.75	0.73	0.76	–	–	–	–	–	–	–	–	–	–	–	–	–	–	–
Rhizomucor pusillus	0.73	0.73	0.73	0.73	−0.73	−0.75	–	–	–	–	–	–	–	–	–	–	–	–	–
Thermomyces lanuginosus	0.73	0.73	0.75	0.73	−0.73	−0.76	–	–	–	–	–	–	–	–	–	–	–	–	–
Wallemia sebi	0.73	0.74	0.76	0.73	–	–	–	–	–	–	–	–	–	–	–	–	–	–	–
Lichtheimia corymbifera	–	–	–	–	–	–	–	–	–	–	–	–	–	–	–	–	–	–	–
Pichia kudriavzevii	–	–	–	–	–	–	–	–	–	–	–	–	–	–	–	–	–	–	–
**DQ biomarkers**																			
Saccharomycopsis fibuligera	–	–	–	–	0.73	0.73	−0.76	−0.73	−0.75	−0.73	−0.75	−0.75	−0.75	−0.73	−0.75	−0.73	−0.75	−0.75	−0.75
Mucor indicus	–	–	–	–	0.73	0.76	−0.73	−0.75	−0.73	−0.75	−0.73	−0.73	−0.73	−0.76	−0.73	−0.75	−0.73	−0.73	−0.73
**AQ biomarkers**																			

Lichtheimia ramosa	–	–	–	–	−0.73	−0.75	0.75	0.73	0.76	0.73	0.76	0.73	0.73	0.73	0.73	0.73	0.73	0.73	0.76
**LQ biomarkers**																			
Rhizopus microspores	–	–	–	–	–	–	–	–	–	–	–	–	–	–	–	–	–	–	–
Saccharomyces cerevisiae	−0.73	−0.73	−0.73	−0.73	–	–	–	–	–	–	–	–	–	–	–	–	–	–	–
**Non–biomarkers**																			
Aspergillus niger	–	–	–	–	–	–	–	–	–	–	–	–	–	–	–	–	–	–	–
Aspergillus penicillioides	0.82	0.81	0.82	0.82	–	–	–	–	–	–	–	–	–	–	–	–	–	–	–
Rhizomucor miehei	–	–	–	–	–	–	−0.82	–	–	–	–	–	–	–	–	–	–	–	–
Cyberlindnera fabianii	−0.75	−0.76	−0.76	−0.75	–	–	–	–	–	–	–	–	–	–	–	–	–	–	–
Diutina rugosa	–	–	–	–	–	–	−0.73	–	–	–	–	–	–	–	–	–	–	–	–
Saccharomycopsis malanga	–	–	–	–	0.90	0.90	–	–	–	–	–	–	–	–	–	–	–	–	–
Syncephalastrum racemosum	–	–	–	–	–	–	–	–	–	–	–	–	–	–	–	–	–	–	–
Clavispora lusitaniae	–	–	–	–	–	–	–	–	–	–	–	–	–	–	–	–	–	–	–
Millerozyma farinosa	0.75	0.76	0.78	0.75	–	–	–	–	–	–	–	–	–	–	–	–	–	–	–
Syncephalastrum monosporum	–	–	–		–	–	–	–	–	–	–	–	–	–	–	–	–	–	–

Note: −, |r| < 0.7 or $p \geq 0.5$ or no correlation.
